# Body Condition Score Estimation Based on Regression Analysis Using a 3D Camera

**DOI:** 10.3390/s20133705

**Published:** 2020-07-02

**Authors:** Thi Thi Zin, Pann Thinzar Seint, Pyke Tin, Yoichiro Horii, Ikuo Kobayashi

**Affiliations:** 1 Graduate School of Engineering, University of Miyazaki, 1 Chome-1 Gakuenkibanadainishi, Miyazaki 889-2192, Japan; pantenzasein.t9@cc.miyazaki-u.ac.jp (P.T.S.); pyketin11@gmail.com (P.T.); 2 Center for Animal Disease Control, University of Miyazaki, 1 Chome-1 Gakuenkibanadainishi, Miyazaki 889-2192, Japan; horii@cc.miyazaki-u.ac.jp; 3 Field Science Center, Faculty of Agriculture, University of Miyazaki, 1 Chome-1 Gakuenkibanadainishi, Miyazaki 889-2192, Japan; ikuokob@cc.miyazaki-u.ac.jp

**Keywords:** body condition score, 3D surface roughness parameters, rotary parlor, 3D camera, regression analysis

## Abstract

The Body Condition Score (BCS) for cows indicates their energy reserves, the scoring for which ranges from very thin to overweight. These measurements are especially useful during calving, as well as early lactation. Achieving a correct BCS helps avoid calving difficulties, losses and other health problems. Although BCS can be rated by experts, it is time-consuming and often inconsistent when performed by different experts. Therefore, the aim of our system is to develop a computerized system to reduce inconsistencies and to provide a time-saving solution. In our proposed system, the automatic body condition scoring system is introduced by using a 3D camera, image processing techniques and regression models. The experimental data were collected on a rotary parlor milking station on a large-scale dairy farm in Japan. The system includes an application platform for automatic image selection as a primary step, which was developed for smart monitoring of individual cows on large-scale farms. Moreover, two analytical models are proposed in two regions of interest (ROI) by extracting 3D surface roughness parameters. By applying the extracted parameters in mathematical equations, the BCS is automatically evaluated based on measurements of model accuracy, with one of the two models achieving a mean absolute percentage error (*MAPE*) of 3.9%, and a mean absolute error (*MAE*) of 0.13.

## 1. Introduction

Tracking body condition scores (BCS), and using them to avoid rapid fluctuations in body weight during the production cycle, has a positive impact on decision-making in dairy farm management, and makes economic sense. It is also useful for improving milk production, health, and reproduction (pregnancy rate) throughout the production cycle. The resulting improved monitoring provides an opportunity to fine-tune nutrition, and healthcare more generally. Although various methods are available for evaluating body condition, many producers use the BCS system, which ranks cattle using an arbitrary scale, and does not rely on body weight [1]. The BCS is assigned by scoring the amount of fat that is observed on several skeletal parts of the cow. Various scoring systems are used to arrive at the BCS, which are used to assign a number as the score. As the system most commonly used, the BCS ranges from 1 to 5, in increments of 0.5 or 0.25. Very thin cows are given a BCS of ‘1’, and very obese cows are rated as ‘5’. The intermediate stages of BCS can be characterized as thin, ideal and obese. A ‘very thin’ cow has prominent hips and spine. The hips and spine of a ‘thin’ cow are easily felt without pressure, and those of an ‘ideal’ cow can be felt with firm pressure. A ‘very obese’ cow is heavily covered by fat. BCS ‘3’ is considered ideal. Cows can then be managed and fed according to the requirements for attaining an optimal BCS.

Research results indicate that optimizing BCS can positively influence the health and productivity of dairy cows. In addition, a rapid decrease in the BCS after calving closely correlates with metabolic disorders and other problems [2]. Current interpretations of available evidence indicate that metabolic disorders affect the immune system of dairy cows during the critical transition from calving [3]. BCS decreases during the approximate 100-day period from calving through early lactation to peak milk, and then increases through dry-off. Generally, maintaining an optimal BCS is needed to avoid extremes of too fat or too lean [4]. For a proper evaluation of BCS, the observer must be familiar with skeletal structures and fat reserves, as described in [5]. In the measurement of BCS using vision-based technology, tailhead and loin areas are of primary concern. Many researchers have evaluated BCS by manually checking off significant anatomical points on digital 2D images. Hook angles and tailhead depressions are formulated to estimate BCS, using a technique introduced in 2008 by Bewley et al. [6]. In this technique, the skeletal checkpoints associated with anatomical structures are used in the assessment of BCS. However, automating the identification of these checkpoints with 2D images is difficult. A new perspective for measuring fat levels in cows is proposed using ultrasonography in [7]. It shows that the larger the BCS, the more the increase in the fat reserves. In recent years, single 3D camera and multiple 3D cameras with multiple viewpoints have been introduced to evaluate the body condition score by using machine vision technology. In our system, we introduce the BCS automation system by using a single 3D camera that is mounted above the rotary parlor.

## 2. Related Work

To rate BCS, a technique was introduced by Edmonson et al. in [8], which consists of manually assessing the amount of body fat around the tailhead, as well as by palpation of the tailhead (the depression beneath the tail), and the pelvis (hook and pin bones). In an automated system, an image analysis technique was introduced to derive relevant characteristics from anatomical points, and from intensities or depth values in regions of interest and cow contours. By using a low-cost 3D camera, an automatic body condition scoring system was developed by implementing an image-processing technique and regression algorithms [9]. In this system, fourteen features correlated with BCS were used (such as age, weight, and height), including some features that were derived from video images, and automatically derived from farm records. The accuracy of the entire system was 0.26 of mean absolute error (*MAE*). In [10], an automated BCS rating system was introduced, which assesses scores from 1.5 to 4.5 by extracting multiple features related to body condition from three viewpoints. In this system, body images are recorded using 3-dimensional cameras positioned above, behind, and to the right. Anatomical landmarks are automatically identified, and then bony prominences and surface depressions are quantified to evaluate BCS and provide the result.

In our own previous work, we proposed a noninvasive method for automatically evaluating BCS [11]. This method starts with a 3D image, from which two analytical models are created, one using the root-mean-square deviation (RMSD), and the other using the convex hull volume parameter. This method resulted in a standard error of 0.35 using RMSD, and 0.19 using convex hull volume. We also noticed that convex hull volume has a strong correlation with BCS. The benefits of continuously monitoring BCS are intuitive to most dairy producers, nutritionists, and others involved in dairy farming. A few dairy farms have incorporated such monitoring as part of their management strategy, as described in [12]. In our previous paper of body condition indicators described in [13], we noted a strong link between BCS and parameters such as convex hull volume and mean height, with BCS ratings between 3.5 and 3.75. That system also introduced variations in BCS trends during the calving and lactation intervals using values for monthly mean height. In [14], a low-cost monitoring system was proposed for unobtrusively and regularly monitoring BCS, lameness, and weight using 3D imaging technology. In the paper described in [14], a new approach for assessing BCS based on a rolling ball algorithm was validated by achieving repeatability within ±0.25 BCS. Our approach included automatic image selection steps for each cow in the parlor that was targeted for a smart application of continuous monitoring. The approach also featured a newly developed BCS estimation model using two region of interests (ROI) visible from above. Finally, this approach also involves extracting 3D surface roughness features, and generalizing two linear regression models to estimate BCS by applying the proposed parameters. 

## 3. Data Collection and Preprocessing

**Figure 1:**
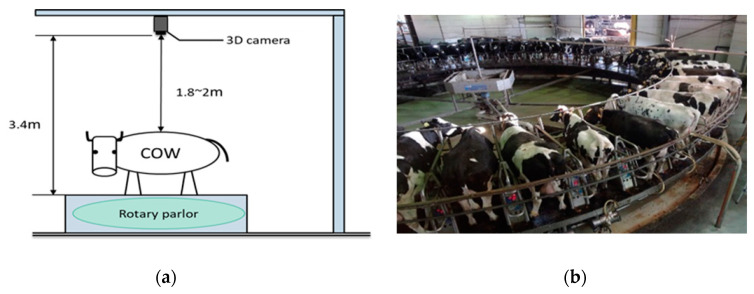
(**a**) Position of 3D camera; (**b**) image of cows in rotary parlor.

In our proposed system, a 3D camera is mounted 3.4 m above a rotary parlor. Data collection is done at a large-scale dairy farm in Oita Prefecture, Japan. The position of the 3D camera and an illustration of cows in the parlor are seen in Figure 1a,b. The 3D camera generates a resolution of 132 × 176 pixels in X, Y, and Z directions. X and Y are the x and y coordinates of the image and Z is the distance information (z or D_0_) of the image. 

**Figure 2:**
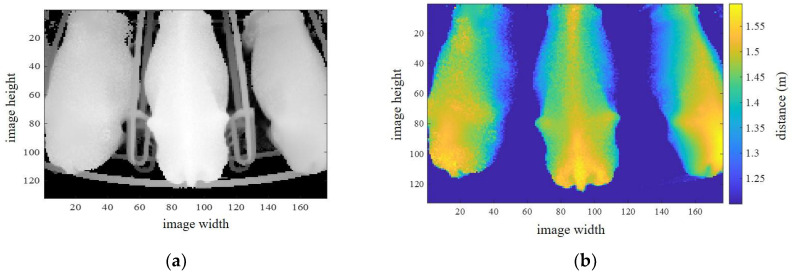
Cow region extraction from 3D camera. (**a**) Original image in rotary parlor from 3D camera; (**b**) Cow region extraction by distance information.

**Figure 3:**
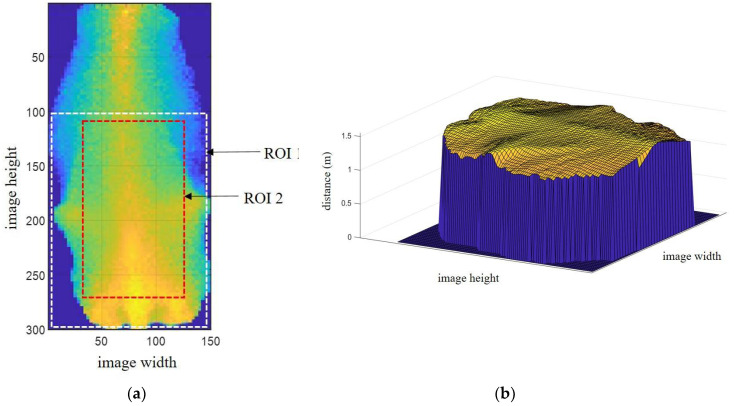
The processed cow image. (**a**) Distance image of cow (color expresses the distance); (**b**) The cow image in 3D space.

The proposed system uses a single 3D camera and generates data in csv format (comma-separated values). Each line of the csv data has 23,232 values for distance information, which is preprocessed into image dimensions of 132 × 176 pixels. The transformed image has a maximum of three cows. An original image obtained using this camera is shown in Figure 2a. The original image shows the distance data (D_0_) from the camera center to the image plane. To obtain real-world data for the distance from the ground, the difference between D_0_ and the camera height (3.4-D_0_) m is calculated. The distance range between 1.21 m and 2.1 m is considered to be the cow region, as shown in Figure 2b. Conversely, the background region is automatically removed by the extraction of the cow region. Each of the cows has their related ID number, using radio frequency identification (RFID). Therefore, we only extract the middle cow image as the desired ID number on the rotary parlor, as shown in Figure 3a,b. The sided images of other ID numbers are conversely removed. In Figure 3a, ROI 1 and ROI 2 are the two regions of interest used for BCS estimation, for which details are discussed in Section 4.

## 4. Automatic Image Selection Process by Filtering

**Figure 4:**
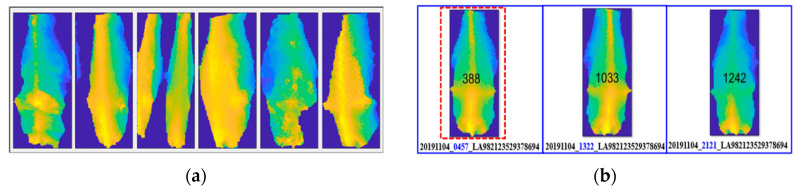
Sample discarded and filtered images. (**a**) Sample of discarded images; (**b**) Image selection by symmetricity.

Sometimes, the 3D camera returns distorted images. These bad images are removed using geometric and hole areas. A sample of images discarded due to distortion and touching between cows is shown in Figure 4a. The selected cow images are grouped by ID number. From all of the cow images recorded, filtering ensures that only one good position of cow image for the same ID number on the same day is selected by using the symmetricity parameter, though the camera generally captures three images per day in the milking parlor. Filtering is performed by a comparison of symmetricity. The filtering for cow ID “LA982123529378694” is shown in Figure 4b. This cow has camera capture times (at 04:57 a.m., 13:22 p.m., and 21:21 p.m. on 4th November 2019), and the selected filtered image is shown by a red marker. When the left and right sides of the image are symmetric, the difference between the two areas is nearly zero. The image with the least difference in value is selected as the most symmetric image, filtered from the images collected every day.

**Figure 5:**
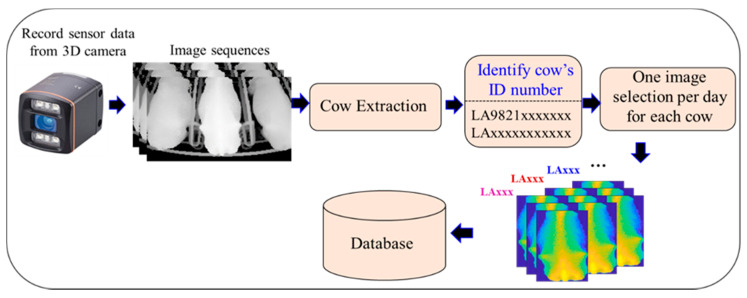
Detailed workflow for automatic image selection.

A detailed workflow for the automatic image selection process by filtering is shown in Figure 5. After making the cow extraction with the filtering step, each of the selected or filtered images is stored by its ID group in the database for further implementation of the smart system. In our system, approximately 20,000 images were automatically discarded by geometric area as bad images, and over 140,000 selected images were recorded from August 2018 to February 2020.

The proposed work is performed on Windows 10, an Intel ^®^ Core ™ i7-7700 CPU, @ 3.6 GHz. The processing time for the cow selection process from each set of csv data is approximately 0.2 s. BCS is a good management tool for developing nutrition and care programs for specific situations. This is the first step in improving the use of BCS. Follow-on steps include developing a BCS monitoring program for each individual cow, determining the BCS at calving, and then monitoring changes in BCS during lactation. Optimal scores can be devised for each cow at each stage of the production cycle, i.e., the optimal score for calving is 3.25 and the optimal score at the start of breeding is 3, and so on. Therefore, we launched this study to automate an accurate assessment of BCS in the next step of BCS modeling.

This section includes a discussion of automatic collecting of cow images from a 3D camera. The remaining sections of the paper include a discussion of collecting cow images and their use in building models in Section 4, experimental use and performance evaluation of the two analytical models in Section 5, and a presentation of conclusions, as well as prospective work, in Section 6. 

## 5. Proposed BCS Modeling

BCS is generally evaluated by experts who have been trained in its use. Though this conventional method is time- and labor-intensive, automation can ease the burden. Using manual measurements for BCS as a baseline or for referencing a model, a reliable automated system can be established. To confirm the performance of the proposed system, we used images of cows with manually measured BCS values in the range of 2.5 to 4. These cow images were collected from two different farms: (1) the Sumiyoshi Livestock Science Station, Field Science Center, University of Miyazaki, and (2) a large-scale dairy farm in Oita Prefecture, Japan. Two experts performed these initial manual measurements. Although the possible BCS scores range from 1 to 5 for very thin to very obese cows, respectively, BCS values between 2.5 and 4 are the most frequently seen. Our BCS dataset is shown in Table 1. In order to evaluate BCS values, two analytical models (*M*1 and *M*2) are proposed for the automated system. In total, 52 cows were used in the experiment, 32 for training, and an additional 20 for testing. *M*1 was applied to ROI 1, and *M*2 was applied to ROI 2, as seen in Figure 3a. The learning parameters were extracted for the two regions of interest (ROI 1 and ROI 2) using the concept of 3D roughness texture, which can be seen in surface texture analysis ASME-B46.1 (American Society of Mechanical Engineers 2002).

### 5.1. BCS Estimation Model 1

**Figure 6:**
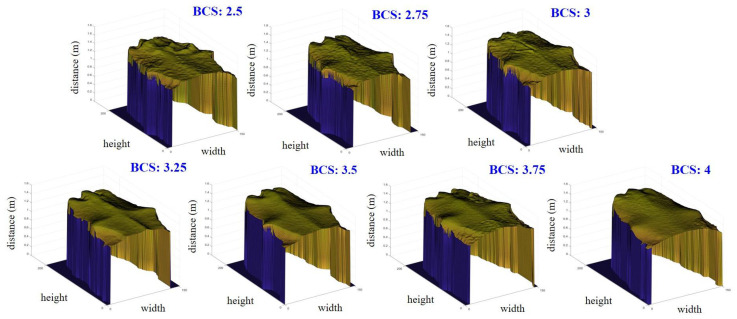
Images composed of cross-sectional slices for ROI 1 used in BCS estimation model 1.

In the 3D image of a cow’s backbone, BCS estimation model 1 was used for ROI 1, which is two-thirds of the whole cow body starting from the tailhead, as seen in Figure 3a. Variations in the amount of fat reserves or in the energy balance are apparent in that region. Moreover, the more that body fat covers the bones, the larger the BCS. Visually, we can clearly differentiate thin cows from fat ones by this coverage by fat. Therefore, roughness parameters are extracted to determine the BCS. Figure 6 shows images composed of cross-sectional slices in ROI 1 for each BCS value between 2.5 and 4 in increments of 0.25. In this proposed region, the following parameters were extracted:

- Arithmetic mean height (*A*_1_);
- Convex hull volume (*A*_2_);
- Difference between convex hull volume and 3D volume (*A*_3_);
- Difference between peak height and valley depth in fifteen maximums and minimums for all profiles (*A*_4_).

Arithmetic mean height is calculated by following Equation (1):

(1) $$\mathrm{Arithmetic}\;\mathrm{mean}\;\mathrm{height}\;=\bar{z}=\frac{1}{N}\sum_{i=1}^{N}z_{i}$$

where *z* is the height or distance parameter on the roughness profile, and *N* is the total number of all height values in ROI 1.

Convex hull volume is calculated by [15]:

(2) $$Convex\;hull\;volume=\frac{1}{3}\times \sum_{F}\left(height\times area\;of\;face\right)$$

where *F* represents the faces of the polyhedron.

The difference between peak height and valley depth in fifteen maximums and minimums is calculated by using Equation (3):

(3) $$\mathrm{Difference}\;\mathrm{between}\;\mathrm{peak}\;\mathrm{and}\;\mathrm{valley}\;\mathrm{points}\;=\frac{1}{15}\sum_{i=1}^{15}z_{i}-\frac{1}{15}\sum_{j=1}^{15}z_{j}$$

By using *A*_1_, *A*_2_, *A*_3_, and *A*_4_ features, a stepwise linear regression model (*M*1) is generated by the following Wilkinson notation:

(4) M1~1+A3+A1 ∗A2+A2 ∗A4+A1^2+A4^2

where *M*1 is the BCS obtained by proposed method 1.

### 5.2. BCS Estimation Model 2

**Figure 7:**
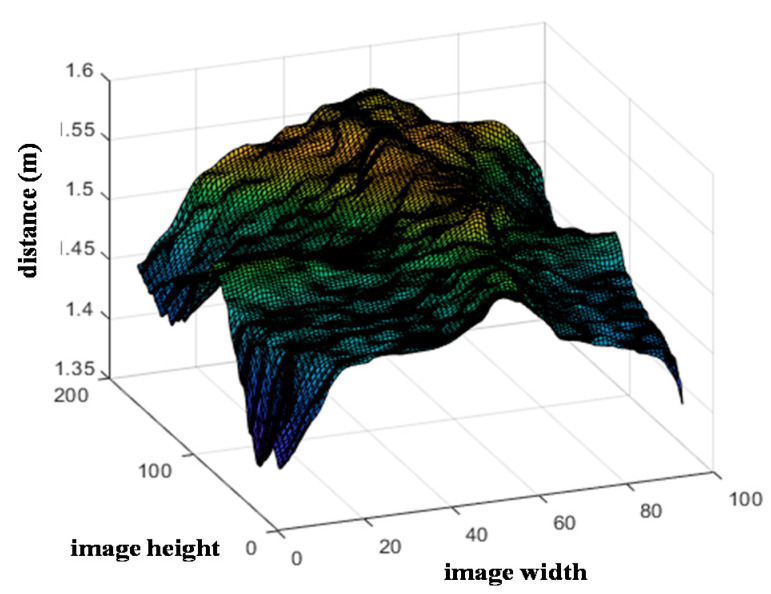
Image composed of cross-sectional slices for ROI 2 used in BCS estimation model 2.

To build BCS estimation model 2, ROI 2 in Figure 3a was used, for which an image composed of cross-sectional slices, is shown in Figure 7. In this region, the following features are extracted:

- Arithmetic mean height (*B*_1_);
- Difference between peak height and valley depth (*B*_2_).

The stepwise linear regression model (*M*2) was generated using two learning parameters (*B*_1_ and *B*_2_). The output of the second proposed BCS model is defined using Wilkinson notation, as seen in Equation (5).

(5) M2~1+B1∗ B2+B2^2

where *M*2 is the BCS obtained by proposed method 2.

Stepwise linear regression is a semi-automated process used for building a model. It can be generated as a way of adding or removing predictor parameters. Parameters or features to be added or removed are picked up from statistics on the test of estimated coefficients used to reach the target output.

In the selected cow image, the BCS estimation model is established after the parameter extraction. The processing time to obtain the BCS output is about 0.13 s. The results for training and testing BCS measurements for the two proposed models are seen in Table 2.

## 6. Performance Evaluation

For automating BCS estimation, we proposed two analytical models, each used for a 3D image of the top view of the region of interest (ROI). The analytical parameters were extracted from pixel depth values. The training models were built using data collected on 32 cows, including one cow for each of the BCS scores of 2.5, 2.75, and 4, four cows for BCS 3, fourteen cows for BCS 3.25, eight cows for BCS 3.5, and three cows for BCS 3.75. In this experiment, training data were collected between BCS 2.5 and 4. A total of 20 cows were used to determine the accuracy of the models, including two cows for BCS 3, ten cows for BCS 3.25, six cows for BCS 3.5, and two cows for BCS 3.75. Manual assessments of BCS were performed by experts, and these assessments were compared with the results of using the proposed models. The measurable parameters were mean absolute error (*MAE*), and mean absolute percentage error (*MAPE*). Performance evaluations for models 1 and 2 are shown in Table 3. According to test results, the mean absolute error percentage for model 2 (*M*2) was less than that for model 1 (*M*1); i.e., small error values indicate good predictive capability. Calculations for *MAE* and *MAPE* were performed using Equations (6) and (7):

(6) $$MAPE=\frac{100\%}{n}\Sigma \left|\frac{y-y_{i}}{y}\right|$$

(7) $$MAE=\frac{1}{n}\Sigma \left|y-y_{i}\right|$$

where *n* = the number of cows tested, y = BCS by experts, $y_{i}$ = BCS by the proposed method.

## 7. Discussion and Conclusions

On large-scale dairy farms, management using manual labor is impractical for numerous reasons. Therefore, automated systems of farm management have been a big focus of dairy farmers. Automated milking robots have recently been introduced to replace manual labor, which is time-intensive and requires one-on-one attention to each cow. The nutritional status of each cow can affect milk production. As one of the most important tools for evaluating nutritional status, BCS is a frequent topic of research. Accurately assessing BCS and regularly monitoring BCS trends have become critical requirements. Our belief is that more and more dairy producers will implement automated BCS evaluation as an important part of smart dairy farming.

The initial step in the proposed system involves automatically selecting images for individual cows. We have tested this process by collecting images in various groups to implement in the advance application. The processing time for this proposed work is acceptable for large-scale data sources. In the next step, automated BCS estimation models are introduced by combining proposed parameter extraction and the two stepwise regression analysis to evaluate BCS. Since our dataset contains only one cow each for BCS of 2.5, 2.75, and 4, we could not test for these BCS in our testing process although they are used in the training for having a good estimation model. Our proposed regression models are tested on 20 cows of BCS (3, 3.25, 3.5, and 3.75). The first model obtains minimum and maximum errors of 0.0025 and 0.45, and the second model obtains minimum and maximum errors of 0.0024 and 0.55, respectively. To measure the proposed model accuracies, the mean absolute parameter (MAE) is used and the MAE for the first and second models was 0.15 and 0.13, respectively. Although the proposed stepwise regression worked reasonably well for developing useful models in this existing data, it cannot always work well for all new data. Therefore, our future work entails collecting more fruitful data of various BCS for the purpose of discerning variations in our proposed automated BCS evaluation process.

## Figures and Tables

**Table 1:** BCS dataset taken by experts.

**Body Condition Score (BCS)**	2.5	2.75	3	3.25	3.5	3.75	4
**No. of Cows**	1	1	6	24	14	5	1

**Table 2:** Comparison of BCS obtained manually by experts, and BCS obtained by proposed models 1 and 2.

Training with Two Proposed Methods by Regression Analysis				Testing with Two Proposed Methods by Regression Analysis			
Cow No.	BCS by Experts	BCS by Proposed Method (Model 1)	BCS by Proposed Method (Model 2)	Cow No.	BCS by Experts	BCS by Proposed Method (Model 1)	BCS by Proposed Method (Model 2)
1	3	3.39	3.32	1	3	3.29	3.27
2	3.5	3.31	3.35	2	3.25	3.34	3.35
3	3.25	3.35	3.64	3	3.25	3.14	3.29
4	3.25	3.28	3.56	4	3.25	3.39	3.28
5	3.25	3.29	3.30	5	3.25	2.79	3.36
6	3.25	3.54	3.29	6	3.5	3.47	3.53
7	3	3.21	3.12	7	3.25	3.40	3.24
8	3.25	3.20	3.07	8	3.5	3.77	3.36
9	3	3.09	3.34	9	3.5	3.40	3.43
10	3.25	3.10	3.29	10	3.5	3.37	3.31
11	3.5	3.52	3.42	11	3.25	3.28	3.25
12	3.5	3.18	3.06	12	3.5	3.34	3.18
13	3.5	3.59	3.28	13	3.25	3.44	3.28
14	3.25	3.10	3.17	14	3.5	3.27	3.50
15	3.25	3.48	3.21	15	3.75	3.59	3.20
16	3.5	3.38	3.51	16	3.75	3.52	3.53
17	3.25	3.05	3.12	17	3.25	3.25	3.21
18	3.75	3.73	3.53	18	3	3.04	3.16
19	3.25	3.29	3.30	19	3.25	3.41	3.43
20	3.25	3.20	3.40	20	3.25	3.08	3.38
21	3.25	3.40	3.26				
22	3.5	3.45	3.32				
23	3.5	3.48	3.18				
24	3.25	3.30	3.37				
25	3.75	3.67	3.67				
26	3.25	3.11	3.25				
27	2.5	2.62	2.89				
28	3.5	3.32	3.27				
29	2.75	2.88	3.21				
30	3.75	3.41	3.41				
31	4	3.81	3.61				
32	3	3.28	3.26				

**Table 3:** Performance evaluation for models 1 and 2.

BCS Model	Training		Testing	
	*MAE*	*MAPE*	*MAE*	*MAPE*
Model 1 (*M*1)	0.14	4.31%	0.15	4.64%
Model 2 (*M*2)	0.19	5.89%	0.13	3.87%

## References

[1] Kellogg W. Body Condition Scoring with Dairy Cattle. https://www.uaex.edu/publications/pdf/FSA-4008.pdf.

[2] Gearhart M.A., Curtis C.R., Erb H.N., Smith R.D., Snien C.J., Chase L.E., Cooper M.D. (1990). Relationship of changes in condition score to cow health in Holsteins. J. Dairy Sci..

[3] Roche J.R., Meier S., Heiser A., Mitchell M.D., Walker C.G., Crookenden M.A., Riboni M.V., Loor J.J., Kay J.K. (2015). Effects of precalving body condition score and prepartum feeding level on production, reproduction, and health parameters in pasture-based transition dairy cows. J. Dairy Sci..

[4] Heinrichs A.J. (1980). Body-Condition Scoring as a Tool for Dairy Herd Management.

[5] Rossi J., Wilson T.W. Body Condition Scoring Beef Cows. https://secure.caes.uga.edu/extension/publications/files/pdf/B%201308_3.PDF.

[6] Bewley J.M., Peacock A.M., Lewis O., Boyce R.E., Roberts D.J., Coffey M.P., Kenyon S.J., Schutz M.M. (2008). Potential for Estimation of Body Condition Scores in Dairy Cattle from Digital Images. J. Dairy Sci..

[7] Alapati A., Kapa S.R., Jeepalyam S., Rangappa S.M.P., Yemireddy K.R. (2010). Development of the body condition score system in Murrah buffaloes: Validation through ultrasonic assessment of body fat reserves. J. Dairy Sci..

[8] Edmonson A.J., Lean I.J., Weaver L.D., Farver T., Webster G. (1989). A body condition scoring chart for Holstein dairy cows. J. Dairy Sci..

[9] Spoliansky R., Edan Y., Parmet Y., Halachmi I. (2016). Development of automatic body condition scoring using a low-cost 3-dimensional Kinect camera. J. Dairy Sci..

[10] Song X., Bokkers E.A.M., van Mourik S., Koerkamp P.G., van der Tol P.P.J. (2019). Automated body condition scoring of dairy cows using 3-dimensional feature extraction from multiple body regions. J. Dairy Sci..

[11] Imamura S., Zin T.T., Kobayashi I., Horii Y. Automatic evaluation of cow’s body-condition-score using 3D camera. Proceedings of the 2017 IEEE 6th Global Conference on Consumer Electronics (GCCE).

[12] Krukowski M. Automatic Determination of Body Condition Score of Dairy Cows from 3D Images. https://pdfs.semanticscholar.org/a9e1/bddb0fdc862859b90d03e20b34d4cfdf4b93.pdf.

[13] Zin T.T., Seint P.T., Tin P., Horii Y. The Body Condition Score Indicators for Dairy Cows Using 3D Camera. Proceedings of the International Workshop on Frontiers of Computer Vision (IW-FCV).

[14] Hansen M.F., Smith M.L., Smith L.N., Jabbar K.A., Forbes D. (2018). Automated monitoring of dairy cow body condition, mobility and weight using a single 3D video capture device. Comput. Ind..

[15] Polyhedron. https://en.wikipedia.org/wiki/Polyhedron.

